# Different functional states of fusion protein gB revealed on human cytomegalovirus by cryo electron tomography with Volta phase plate

**DOI:** 10.1371/journal.ppat.1007452

**Published:** 2018-12-03

**Authors:** Zhu Si, Jiayan Zhang, Sakar Shivakoti, Ivo Atanasov, Chang-Lu Tao, Wong H. Hui, Kang Zhou, Xuekui Yu, Weike Li, Ming Luo, Guo-Qiang Bi, Z. Hong Zhou

**Affiliations:** 1 School of Life Sciences, University of Science and Technology of China, Hefei, Anhui, P.R. China; 2 Department of Microbiology, Immunology & Molecular Genetics, University of California, Los Angeles (UCLA), Los Angeles, California, United States of America; 3 Molecular Biology Institute, UCLA, Los Angeles, CA, United States of America; 4 California NanoSystems Institute, UCLA, Los Angeles, CA, United States of America; 5 Department of Chemistry, Georgia State University, Atlanta, GA, United States of America; Institut Pasteur, FRANCE

## Abstract

Human cytomegalovirus (HCMV) enters host by glycoprotein B (gB)-mediated membrane fusion upon receptor-binding to gH/gL-related complexes, causing devastating diseases such as birth defects. Although an X-ray crystal structure of the recombinant gB ectodomain at postfusion conformation is available, the structures of prefusion gB and its complex with gH/gL on the viral envelope remain elusive. Here, we demonstrate the utility of cryo electron tomography (cryoET) with energy filtering and the cutting-edge technologies of Volta phase plate (VPP) and direct electron-counting detection to capture metastable prefusion viral fusion proteins and report the structures of glycoproteins in the native environment of HCMV virions. We established the validity of our approach by obtaining cryoET *in situ* structures of the vesicular stomatitis virus (VSV) glycoprotein G trimer (171 kD) in prefusion and postfusion conformations, which agree with the known crystal structures of purified G trimers in both conformations. The excellent contrast afforded by these technologies has enabled us to identify gB trimers (303kD) in two distinct conformations in HCMV tomograms and obtain their *in situ* structures at up to 21 Å resolution through subtomographic averaging. The predominant conformation (79%), which we designate as gB prefusion conformation, fashions a globular endodomain and a Christmas tree-shaped ectodomain, while the minority conformation (21%) has a columnar tree-shaped ectodomain that matches the crystal structure of the “postfusion” gB ectodomain. We also observed prefusion gB in complex with an “L”-shaped density attributed to the gH/gL complex. Integration of these structures of HCMV glycoproteins in multiple functional states and oligomeric forms with existing biochemical data and domain organization of other class III viral fusion proteins suggests that gH/gL receptor-binding triggers conformational changes of gB endodomain, which in turn triggers two essential steps to actuate virus-cell membrane fusion: exposure of gB fusion loops and unfurling of gB ectodomain.

## Author summary

Infection by herpesviruses leads to many human diseases, ranging from mild cold sores to devastating cancers. Human cytomegalovirus (HCMV) is among the most medically significant herpesviruses and causes birth defects and life-threatening complications in immuno-suppressed individuals. HCMV infection begins with cellular membrane fusion, a dynamic process involving receptor-binding to gH/gL complexes and drastic transformation of fusion protein gB trimer from the metastable prefusion conformation to the stable postfusion conformation. We have used cryo electron tomography incorporating cutting-edge technologies to observe the three-dimensional structures of gB and gH/gL in their native environments of HCMV particles. Visualizations of gB in both prefusion and postfusion conformations, together with structures of other class III viral fusion proteins and molecular dynamics flexible fitting (MDFF), facilitate a prediction for the structure of HCMV gB in its prefusion conformation. With the further observation of the contact of prefusion gB with gH/gL complex, the conformational changes of gB from pre- to postfusion state lead to a better understanding of herpesvirus fusion mechanism.

## Introduction

Human cytomegalovirus (HCMV), a member of the *Betaherpesvirinae* subfamily of the *Herpesviridae* family, is a leading viral cause of birth defects [1, 2] and a major contributor to life-threatening complications in immunocompromised individuals. As one of the largest membrane-containing viruses, HCMV shares a common multilayered organization with all other herpesviruses, composed of an icosahedrally ordered nucleocapsid enclosing a double-stranded DNA genome, a poorly defined tegument protein layer, and a pleomorphic, glycoprotein-embedded envelope [3]. During infection, herpesviruses fuse their envelopes with cell membranes, resulting in the delivery of nucleocapsid into the cytoplasm of the host cells. This complex process requires a number of viral glycoproteins and host receptors functioning in a coordinated manner. Glycoproteins gB and gH/gL are conserved across all herpesviruses and are essential for virus entry into cells [4]. Receptor-binding to gH/gL-containing complexes—the composition of which differs among clinical and laboratory-adapted HCMV strains and across different herpesviruses [5]—triggers conformational changes of fusion protein gB, leading to fusion of the viral envelope with cell membrane [6]. This use of both a fusion protein and a receptor-binding complex for herpesvirus entry differs from many other enveloped viruses, which use a single protein for both receptor binding and membrane fusion.

Averaging up to tens of thousands of particle images by single-particle cryoEM method has resolved *in situ* structures of capsid proteins [7–9] and the capsid-associated tegument protein pp150 [10], up to atomic resolution [11]. However, such method is not applicable to the studies of herpesvirus gB and other glycoproteins due to their disorganized distribution on the pleomorphic viral envelope. Instead, the structures of gB ectodomains and various forms of gH/gL from herpes simplex virus (HSV) [12, 13], Epstein-Barr virus (EBV) [14, 15] and HCMV [16, 17] have been solved by x-ray crystallography. The gB ectodomain structures from these studies share structural similarities to other class III viral fusion proteins in their postfusion conformation [18–20]. Among these proteins, vesicular stomatitis virus (VSV) G is the only one whose ectodomain structure has been solved for both prefusion [21] and postfusion [20] conformations, thanks to its pH-reversibility between the two conformations and amenability to crystallization at both high and low pH conditions. At pH 6.3–6.9 conditions, VSV G has also been observed to exist in monomeric forms both in solution and on virion envelope, possibly representing fusion intermediates [22, 23]. By contrast, the prefusion conformation of herpesvirus gB is metastable and its structure has been elusive (even the recent crystal structure of the full-length HSV-1 gB is also in the postfusion conformation [24]). While cryo electron tomography (cryoET) of HSV-1 virions has revealed glycoproteins on the native viral envelope, poor contrast of cryoET reconstructions makes it difficult to distinguish different glycoprotein structures and conformations [25]. Recent efforts resorted to the use of purified HSV-1 gB-decorated vesicles to visualize the prefusion gB, but its domain assignments have been controversial due to difficulties in interpreting cryoET structures with poor contrast and signal/noise ratio (SNR) [26, 27]. As a result, the mechanism underlying the complex process of receptor-triggered membrane fusion remains poorly understood for not only HCMV, but also for other herpesviruses.

Recently, the technologies of electron-counting [28, 29], energy filter and Volta phase plate (VPP) [30] have significantly improved contrast and SNR of cryoEM images and their combined use in cryoET has led to resolution of two functional states of 26S proteasome in neurons [31]. In this study, we first demonstrated the ability to distinguish prefusion and postfusion conformations of the VSV G trimer (171 kD) *in situ* by employing a combination of VPP, direct electron-counting, energy filtering and subtomographic averaging. Application of the same approach to HCMV virions has allowed us to identify different conformational states of HCMV gB (303 kD) in their native virion environments and to determine the *in situ* structure of prefusion gB at a resolution of ~21 Å. Moreover, we also observed prefusion gB forming a complex with gH/gL *in situ* for the first time. Integration of these structures and knowledge of class III viral fusion proteins has led to a working model of how conformational changes drive membrane fusion during HCMV entry into host cells.

## Results

### Establishing VPP cryoET for obtaining *in situ* prefusion and postfusion structures

**Fig 1:**
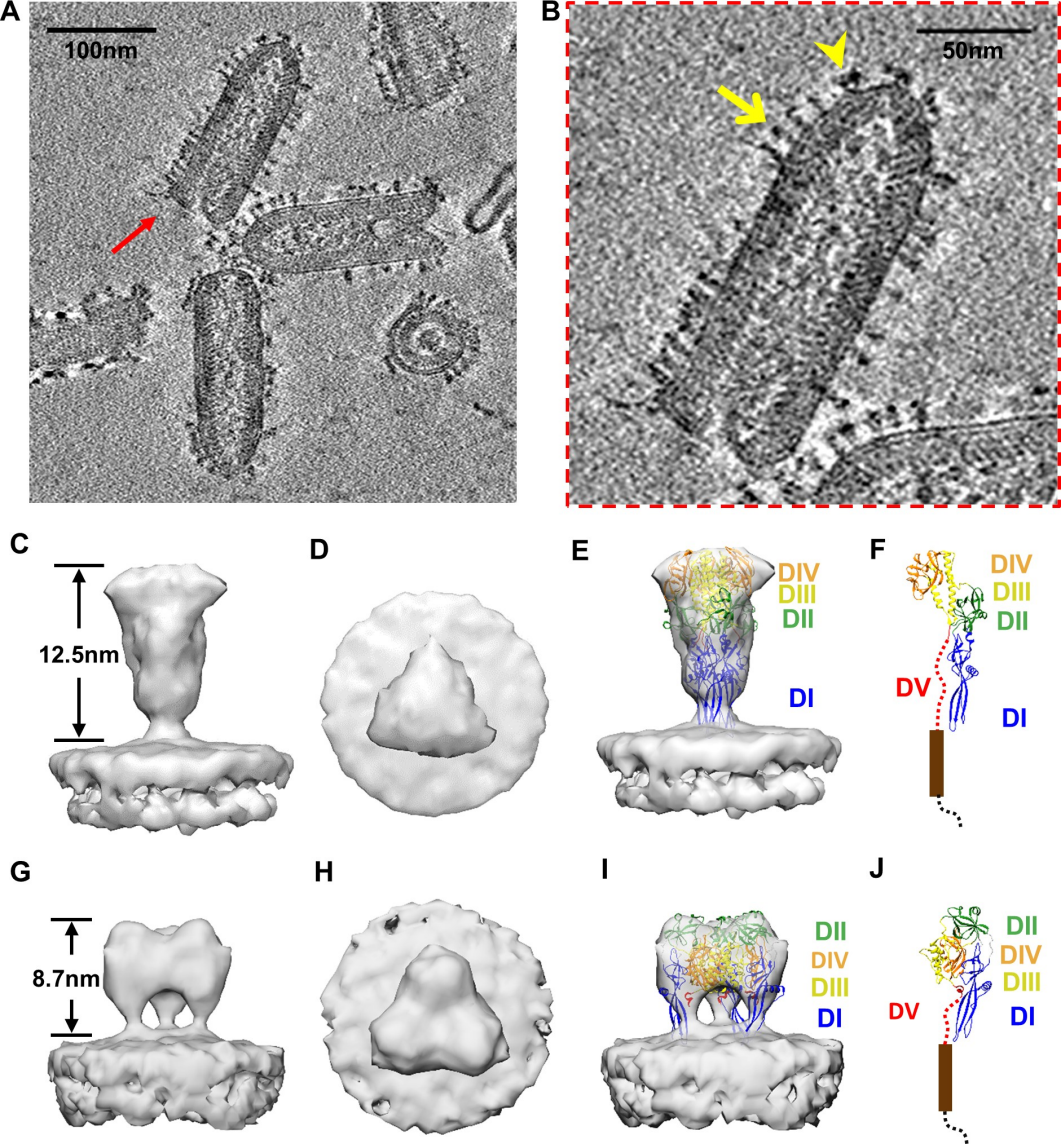
*In situ* structures of two distinct conformations of VSV G. (A) A 10Å-thick slab from the tomogram showing bullet-shaped VSV virions. (B) The VSV particle indicated by red arrow in (A), with a yellow arrow pointing to long-form G and a yellow arrow head pointing to short-form G. (C~F) Subtomographic average of the long-form G densities, whose ectodomain matches the crystal structure of G in postfusion conformation. The subtomographic average of the long-form G densities is shown either as shaded surfaces viewed from side (C) and top (D), or as semi-transparent gray (E) fitted with crystal structure of the G trimer (ribbon) in the postfusion conformation (PDB: 5I2M) [20]. For clarity, one of the subunits is shown alone in (F) with five domains (DI~DV) indicated. (G~J) Subtomographic average of the short-form G densities, whose ectodomain matches the crystal structure of G in prefusion conformation. The subtomographic average of the short-form G densities is shown either as shaded surfaces viewed from side (G) and top (H), or as semi-transparent gray (I) fitted with crystal structure of the G trimer (ribbon) in the prefusion conformation (PDB: 5I2S) [21]. For clarity, one of the subunits is shown alone in (J) with five domains (DI~DV) indicated.

We first established the validity of our cryoET method of combining VPP, direct electron detection, energy filtering, and subtomographic averaging by obtaining *in situ* structures of class III viral fusion proteins with known structures. Towards this end, we took advantage of the relative simplicity of VSV in having a single 57kD glycoprotein, G, on the viral envelope, with its trimeric structures known for both prefusion and postfusion conformations; and used VSV as a gold standard to validate our method. For VSV at pH = 7.5, tomograms reconstructed from tilt series obtained by 300kV Titan Krios equipped with VPP, energy filter and direct electron detection show excellent contrast, enabling the visualizations of G projecting from viral envelope, the helical nucleocapsid, as well as the internal densities corresponding to polymerases L (Fig 1A and 1B). Two conformations of G are readily differentiable based on the height and shape of the ectodomain: the majority is long (12.5nm) and slim, while the minority is short (8.7nm) and fat (Fig 1B). Subtomographic averages of 330 long-form particles and 65 short-form particles from five tomograms both contain a prominent ectodomain, with the long one (~28 Å resolution) fit perfectly with the crystal structure of G ectodomain trimer in the postfusion conformation (Fig 1C–1E) and the short one with that in the prefusion conformation (Fig 1G–1I). Similar structures were observed in a previous electron tomography study performed on negatively stained sample [32].

Both crystal structures of G contain five domains, DI through DV, despite drastic domain arrangements (Fig 1E, 1F, 1I and 1J). The dramatically different appearances between the two conformations are primarily due to the refolding of the short loop (residue 273 to 275) in DIII, resulting in the elongation of the central helix and a taller postfusion trimer. DIII form the trimeric core in both conformations, buried in the center of the cryoET density map (Fig 1E and 1I). The other domains (DI, DII and DIV) undergo a rigid-body type rearrangement—only changing the relative orientations and locations while retaining their domain structures [21] (Fig 1F and 1J). This analysis demonstrates that our cryoET approach incorporating the three cutting-edge technologies can distinguish the two forms of *in situ* structures of glycoprotein G and allows fitting existing domain structures of individual fusion protein into the density maps for functional interpretation.

### Unprecedented structural details revealed by VPP cryoET of HCMV particles

**Fig 2:**
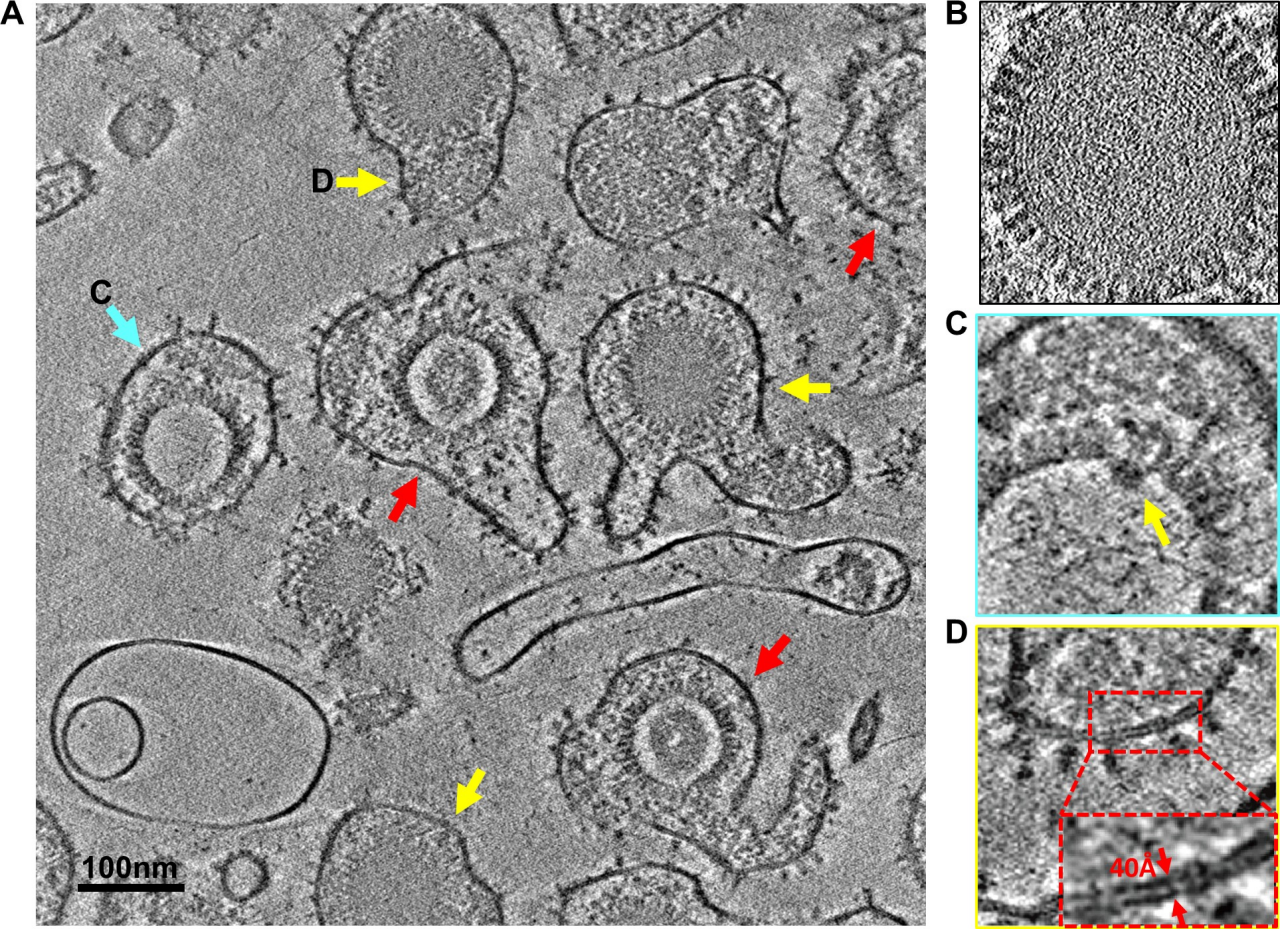
CryoET of HCMV. (A) A 10Å-thick slab from the tomogram of S2 Movie showing a digital slice of various HCMV particles, each comprising an outer envelope layer, an intermediate tegument compartment, and an icosahedral capsid inside. Though all wrapped a pleomorphic glycoprotein-decorated envelope, these particles differ inside: each virion (yellow arrows) containing a C capsid (with DNA genome) and non-infectious enveloped particle (NIEP) either an A capsid (empty, blue arrow), or a B capsid (containing scaffolding protein but no DNA, red arrows). (B) A zoom-in slice of a C capsid, with dsDNA duplexes resolved among the fingerprint-like pattern of the genome. (C) A slice from the particle indicated by the cyan arrow in (A) showing the unique portal complex (arrow) at one of the 12 vertices. (D) A zoom-in envelope region of the particle indicated by a yellow arrow in (A) showing the two resolved leaflets of the lipid bilayer envelope (inset: the enlarged boxed region). The side of the boxes in B-D is 120 nm.

**Fig 3:**
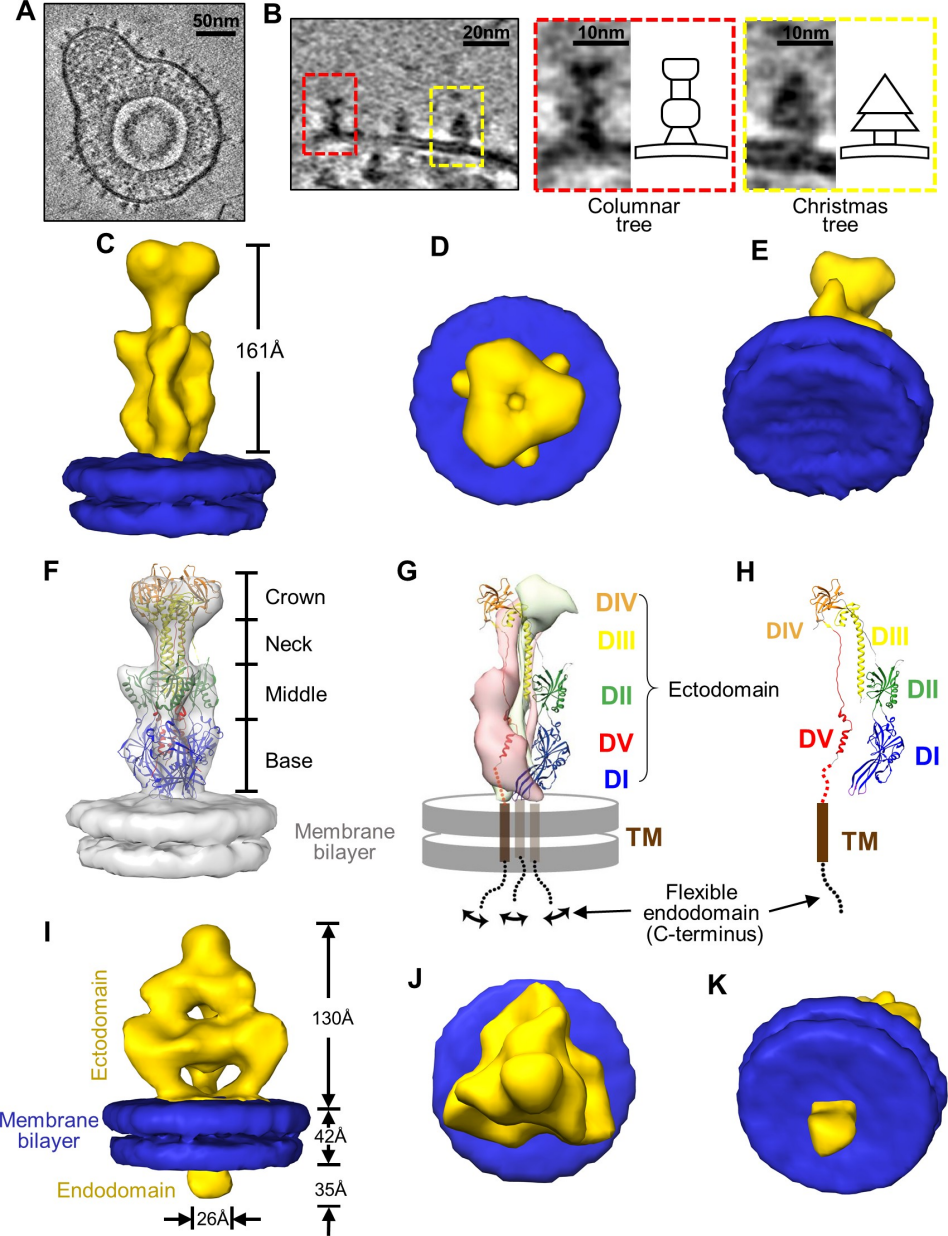
*In situ* structures of gB in the “postfusion” and prefusion conformations. (A) A representative virion showing various glycoprotein densities on its envelope. (B) Identifications of the columnar tree-shaped (red box) and Christmas tree-shaped (yellow box) glycoprotein densities that both match the expected volume of gB (see main text). Insets are the enlargements of the two forms with their corresponding shape schematic. (C~H) Sub-tomographic average of the columnar tree-shaped glycoprotein densities, whose ectodomain matches the crystal structure of gB ectodomain in the postfusion conformation (PDB: 5CXF) [16]. The subtomographic average of the columnar-shaped densities (yellow) and segmented membrane bilayer (blue, from I) are shown either as shaded surfaces viewed from side (C), top (D) and slanted bottom (E), or as semi-transparent gray fitted with the gB ectodomain trimer crystal structure (ribbon) at the postfusion conformation (PDB: 5CXF) [16] (F). Two subunits of the gB trimer crystal structure are shown as pink and gray surfaces, while the third subunit as ribbons with its domains colored as in [16] and its transmembrane helix as brown cylinder and the C-terminal flexible endodomain as a swinging dotted lines (G). For clarity, the third subunit is shown alone in (H) with five domains (DI~DV) indicated. (I~K) Sub-tomographic average of the Christmas tree-shaped densities (yellow) and associated membrane bilayer (blue) viewed from side (I), top (J) and slanted bottom (K).

Next, we applied the same strategy established above to obtain *in situ* structures of gB and its interaction with gH/gL complex. We imaged virions of the highly passaged laboratory HCMV strain AD169, taking advantage of its simplicity, as it has lost some glycoprotein genes and does not contain gH/gL/UL128/UL130/UL131A pentamers on its envelope [33]. We recorded cryoET tilt series of HCMV virions with and without VPP in a Titan Krios instrument equipped with an energy filter and a direct electron detector in electron-counting mode. Both the raw images in the tilt series and the reconstructed tomograms show significantly better contrast when VPP was used (S1 Fig, S1–S4 Movies). Typical in virions obtained by high-speed centrifugation, the viral envelopes are pleomorphic and often exhibit membrane blebs likely due to mechanical stress during purification (Fig 2A). [As discussed below, such mechanical stress might also be responsible for triggering some of the “spring-loaded”/higher-energy (prefusion) gB to its lower energy (“postfusion”) form, which were used as an internal control to validate our cryoET subtomographic averaging method.] In the tomograms reconstructed from the tilt series obtained with VPP (referred to as VPP tomograms) (Fig 2), three types of enveloped viral particles are readily recognized: virions with C-capsid containing densely-packed dsDNA genome (Fig 2B), non-infectious enveloped particles (NIEPs) with B-capsid containing a protein scaffold (red arrows in Fig 2A) or with empty A-capsid (cyan arrow in Fig 2A). Inside C-capsids, the dsDNA molecule occupies evenly throughout the entire interior of the capsid with the 20 Å-diameter dsDNA duplex resolved (Fig 2B)—the first time such detailed features ever observed directly by cryoET. In B-capsids, the scaffolding protein (pUL80, up to 1000 copies/capsid [34, 35]) is organized into a density sphere with an outer and inner diameter of ~700 and ~400Å, respectively. In capsids devoid of genome DNA, a portal complex for DNA translocation is visible at one of the 12 vertices of the capsid (Fig 2C). The viral envelope is pleomorphic (Fig 2A) and its membrane resolved into two leaflets 40Å apart (Fig 2D), sporting sparsely and randomly located, and clearly identifiable glycoprotein spikes on the outer leaflet (Fig 2A and Fig 3A).

### Identifications of gB trimers on the viral envelope

We used the following three pieces of evidence to establish the identifications of gB trimers on the viral envelope. First, among HCMV glycoproteins, gB is known to only exist as homotrimer with a combined mass of ~300 kD [36] and is the most abundant complex over 100 kD [37]. This mass is expected to occupy an estimated extracellular volume of ~300 nm^3^. Among the density spikes decorating the outer leaflet of the viral membrane, only two differently shaped spikes with such volume were identified, suggesting that they might be gB trimer at different conformational states (Fig 3B). Second, the two distinctive side-view shapes—one triangular, Christmas-tree like (71%) and the other rectangular, columnar-tree like (29%) (Fig 3B)—are similar to the side-views of the cryoET reconstructions of HSV-1 gB trimers on purified vesicles in their putative prefusion and postfusion conformations, respectively [27]. Third, we performed subtomographic averaging to these two types of spikes, respectively, in order to examine them with a higher SNR. Both of the averaged models exhibit apparent three-fold symmetry with the symmetric axis perpendicular to the plane of viral membrane, despite slight distortion arising from the inherent “missing wedge problem” of electron tomography (S2 Fig). These three pieces of evidence all point to our tentative assignment of the Christmas tree-shaped and the columnar tree-shaped densities on the HCMV envelope as gB trimers in the prefusion and “postfusion” (quotation marks are used here since the conformation is not really *caused* by fusion but likely triggered by mechanical stress during virion purification with high-speed centrifugation) conformations, respectively. Indeed, as shown below, the available crystal structure of gB in the postfusion conformation matches perfectly with our final subtomographic average of the columnar tree-shaped density, further validating our assignments.

### Subtomographic averages of the putative gB in prefusion and “postfusion” conformations

As mentioned above, we performed subtomographic averaging to characterize the two putative gB conformations at a higher resolution. The significantly enhanced contrast afforded by imaging with VPP at a near-focus condition allowed the clear visualizations of different structures in the reconstructed tomograms. For direct comparison, we also obtained tilt series without using a VPP (referred to as non-VPP tomograms). For the latter data, we had to use a significantly larger defocus value (-4μm) to improve image contrast and record much more tilt series (28 total) in order to obtain a similar number of gB particles for subtomographic averaging due to greater difficulties in distinguishing different glycoprotein morphologies in the tomograms (S1 Fig). In addition, the use of large defocus has necessitated correction for contrast transfer function (CTF): the structure obtained without CTF correction contains phase-inverted, incorrect structure information beyond 25 Å (S4E Fig), as reflected by the broken connections between the ectodomain and the viral membrane in the absence of CTF correction (S3B, S4C and S4D Figs).

In total, 350 particles of the columnar tree-shaped and 1509 particles of the Christmas tree-shaped densities were included for subtomographic averaging. For the columnar tree-shaped structure, all particles were extracted from the VPP tomograms due to ambiguities in distinguishing its slender shape from background noise in the non-VPP tomograms. For the Christmas tree-shaped structure, 874 particles, which came from VPP tomograms, were first used and 635 particles from non-VPP tomograms eventually were also included to further improve resolution. Three-fold symmetry was imposed subsequently to improve SNR and the resolution of the averaged structures. Fourier shell correlation (FSC) analyses indicate that the resolutions for the symmetrized 3D subtomographic average of the columnar tree-shaped and Christmas tree-shaped spikes are 26 Å and 21 Å, respectively, based on the gold-standard criterion (S3A Fig).

The subtomographic average of the columnar tree-shaped spike resolves the two leaflets of the bilayer viral envelope and a prominent (161Å in height) ectodomain (Fig 3C–3E, S5 Movie). The ectodomain density matches well with the crystal structure of the HCMV gB ectodomain trimer [16] (Fig 3F–3H), validating our initial assignment of the columnar tree-shaped density as gB structure in its “postfusion” conformation and re-establishing the validity of our approach. The subtomographic average of our putative prefusion gB densities reveals the two leaflets of the bilayer viral envelope with prominent gB densities attached to both: a prominent ectodomain attached to the outer leaflet (130Å in height) and a globular (about 35Å in height and 26Å in width) endodomain to the inner leaflet (Fig 3I–3K). The ectodomain in the putative prefusion gB is shorter than that in the gB “postfusion” conformation and anchors to the membrane with three well-separated densities, forming a tripod (Fig 3I–3K, S6 Movie). Although no crystal structure of prefusion gB is available to fit into our subtomographic average to directly confirm or refute this prefusion gB assignment, it is believed that herpesvirus gB bears structural and mechanistic similarities to other class III viral fusion proteins, which can be used to aid our assignment. Indeed, the postfusion conformation of HCMV gB ectodomain is similar to the postfusion conformations of all other class III viral fusion proteins [18], including the postfusion VSV G (Fig 1C–1F). The lower portion of the prefusion conformation of the VSV G trimer (Fig 1G–1I) has a tripod shape similar to the lower portion of the Christmas tree-shaped density (Fig 3I). The prefusion VSV G trimer is shorter than—and undergoes drastic domain rearrangements towards—its postfusion conformation [20, 21] (Fig 1); likewise, the Christmas tree-shaped density is shorter than the columnar tree-shaped density. Taken together, these characteristic similarities to the prefusion structure of VSV G corroborate our initial assignment of the Christmas tree-shaped density as the *in situ* prefusion structure of HCMV gB trimer.

### Domain assignments of the *in situ* gB structures in both conformations

**Fig 4:**
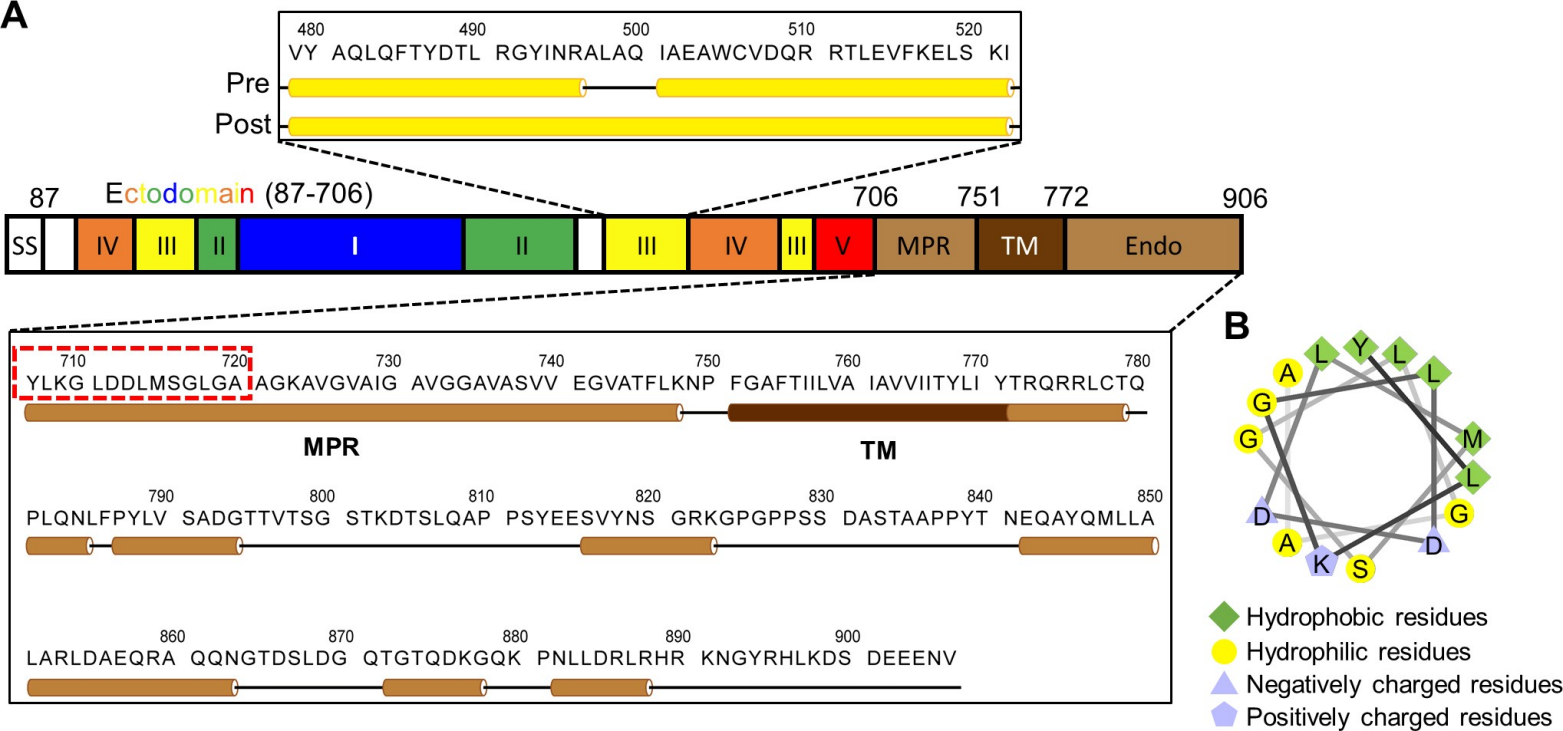
Schematic illustration of the full-length HCMV gB. (A) Mapping of domains to the full-length HCMV gB primary sequence (SS = signal sequence, MPR = membrane proximal region, TM = transmembrane domain, Endo = endodomain). Upper inset: in the prefusion (Pre) conformation, the sequence of the central helix in DIII resolved in the postfusion gB crystal structure (Post) is predicted to fold into two helices joined by a short loop around residues 498–500. Lower inset: predicted secondary structures of the sequence encompassing the MPR, TM and endodomain of gB in the prefusion conformation. (B) Helical wheel diagram of the first 15 amino acids of MPR (sequence in red dashed box in (A)), showing one side with a cluster of hydrophobic amino acids.

Structure-guided sequence analysis (Fig 4A) indicates that each full-length gB protomer contains an N-terminal ectodomain (residues 87–705), a membrane proximal region (MPR, residues 706–750), a single transmembrane helix (residues 751–771) and a C-terminal endodomain (residues 772–906). For the “postfusion” gB trimer, the ectodomain in the subtomographic average can be divided into a base in contact with the membrane, and two lobes—middle and crown—connected by a neck (Fig 3F). The crystal structure of the ectodomain trimer shows that each protomer consists of five domains: DI, DII, DIII, DIV and DV (Fig 3G and 3H) [16]. Except for DV, these domains can be located in our subtomographic average of the “postfusion” gB (Fig 3F). DI, each containing two fusion loops, is located at the base of the trimer; DII and DIV reside, respectively, in the middle and crown lobes, which are connected by DIII in the neck. DV contains a long loop connected by two short helices and is buried, thus is not resolved in our subtomographic average gB trimer due to the limited resolution.

**Fig 5:**
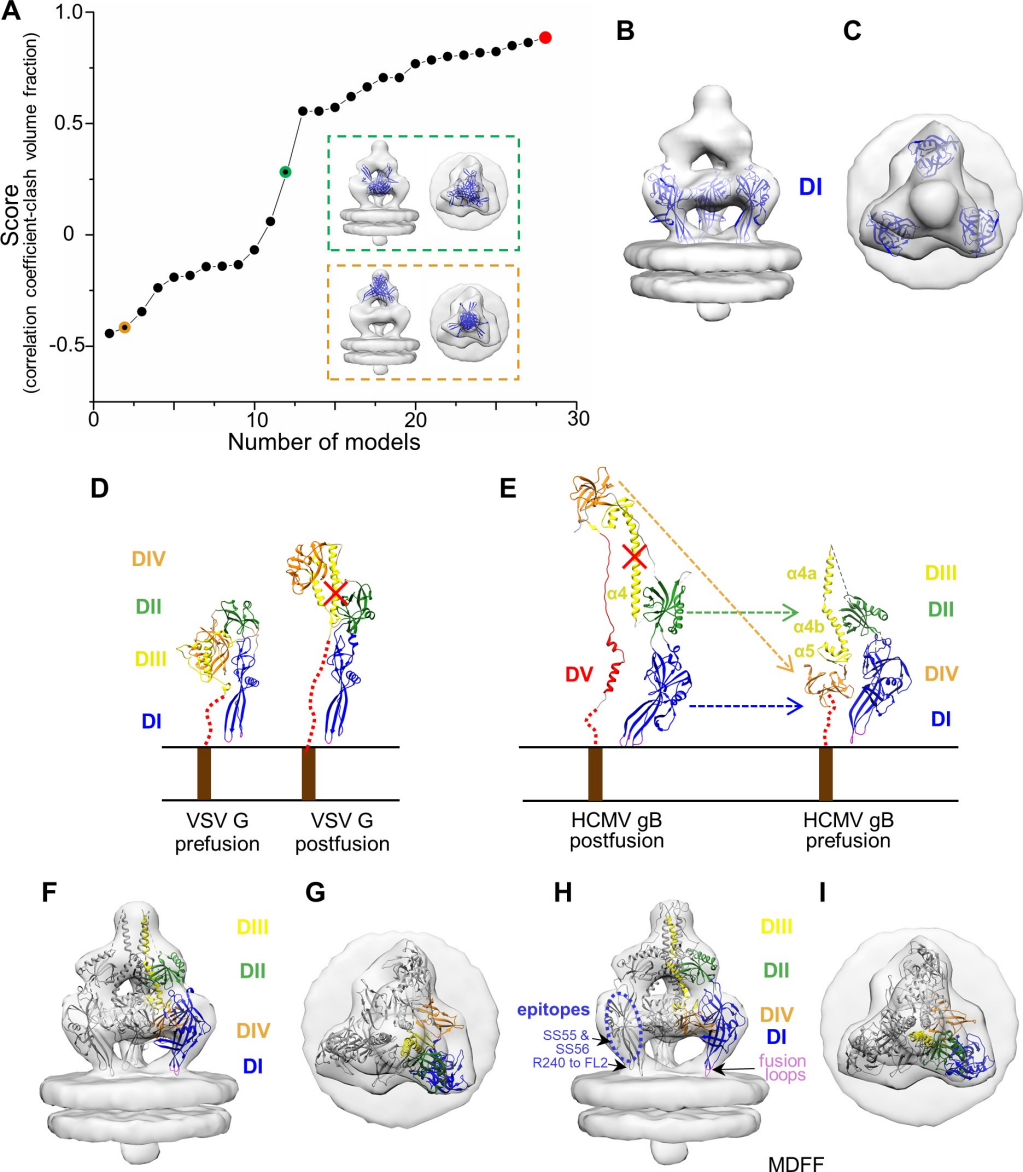
Domain fitting for prefusion gB. (A~C) Domain fitting for DI. (A) Scores of 28 models for DI. Models are ranked in ascending order of scores. Red dot indicates the model of highest score. Dots with green and orange circles indicate the models of medium and low score, respectively. The DI models, superimposed in the subtomographic average of the Christmas tree-shaped density (semi-transparent gray) and viewed from side and top, are shown in green and orange dashed boxes next to the corresponding dots, respectively. (B, C) DI structure, indicated as red dot in (A), is superimposed in the subtomographic average of the Christmas tree-shaped density (semitransparent gray), viewed from side (B) and top (C). (D) VSV G domain rearrangement of crystal monomer structure from prefusion (left, PDB: 2J6J) [21] to postfusion (right, PDB: 2CMZ) [21]. The red dotted lines represent the unresolved domain DV between transmembrane helix and the ectodomain. (E) the crystal structure of one protomer of the postfusion HCMV gB ectodomain (left, PDB: 5CXF) [16] is shown as ribbon next to the predicted prefusion gB structure (right) with domains arranged according to those in the prefusion VSV G. α4 and α5 represent the long central helix and the following short helix in DIII in postfusion gB structure. α4a and α4b represent the two helix breaking from α4. Helices are labeled as in [16]. (F, G) The predicted prefusion gB structure shown in (E, right) is superposed with two other symmetric copies (gray ribbon) in the subtomographic average of the Christmas tree-shaped density (semi-transparent gray), viewed from side (F) and top (G). (H, I) The MDFF-simulated prefusion gB structure is superimposed with two other symmetric copies (gray ribbon) in the subtomographic average of the Christmas tree-shaped density (semi-transparent gray), viewed from side (H) and top (I). The epitopes of HSV-1 antibodies SS55/SS56 and R240 are DI and fusion loop 2 of HSV-1 gB, respectively ([26]); the corresponding locations of these two epitopes in our domain model of HCMV gB are indicated.

As detailed in the Method, we employed a combination of manual rigid-body fitting of known domain structures from the existing HCMV gB postfusion structure [16], comparative modeling of DIII based on the homologous DIII from VSV G prefusion conformation [21], followed by optimization by the molecular dynamics flexible fitting (MDFF) method [38], to put forward a provisional domain arrangement model of the prefusion gB (Fig 5). DV was not considered in our domain modeling of HCMV gB prefusion conformation due to the lack of a template structure, since DV was truncated in the crystal structure of postfusion VSV G. MDFF not only optimized the chemical interactions among the fitted domains, but also improved overall model to map correlation coefficient from 0.83 to 0.94 (Fig 5H and 5I, S7 Movie).

The model from MDFF does not include the MPR (residues 706–750), which is proposed to lie between the ectodomain and the transmembrane helix (Fig 4A) and “mask” the fusion loops to prevent their premature (non-productive) association with lipid [39]. Helical wheel projection of the first 15 amino acids of the MPR shows an amphipathic helix (Fig 4B) whose hydrophobic side could interact with the fusion loops. This notion is consistent with our interpretation of DI in the subtomographic averages of both prefusion and “postfusion” conformations, with the fusion loops pointing to and in close proximity to the membrane.

### Visualization of gB interacting with putative receptor-binding gH/gL complex

**Fig 6:**
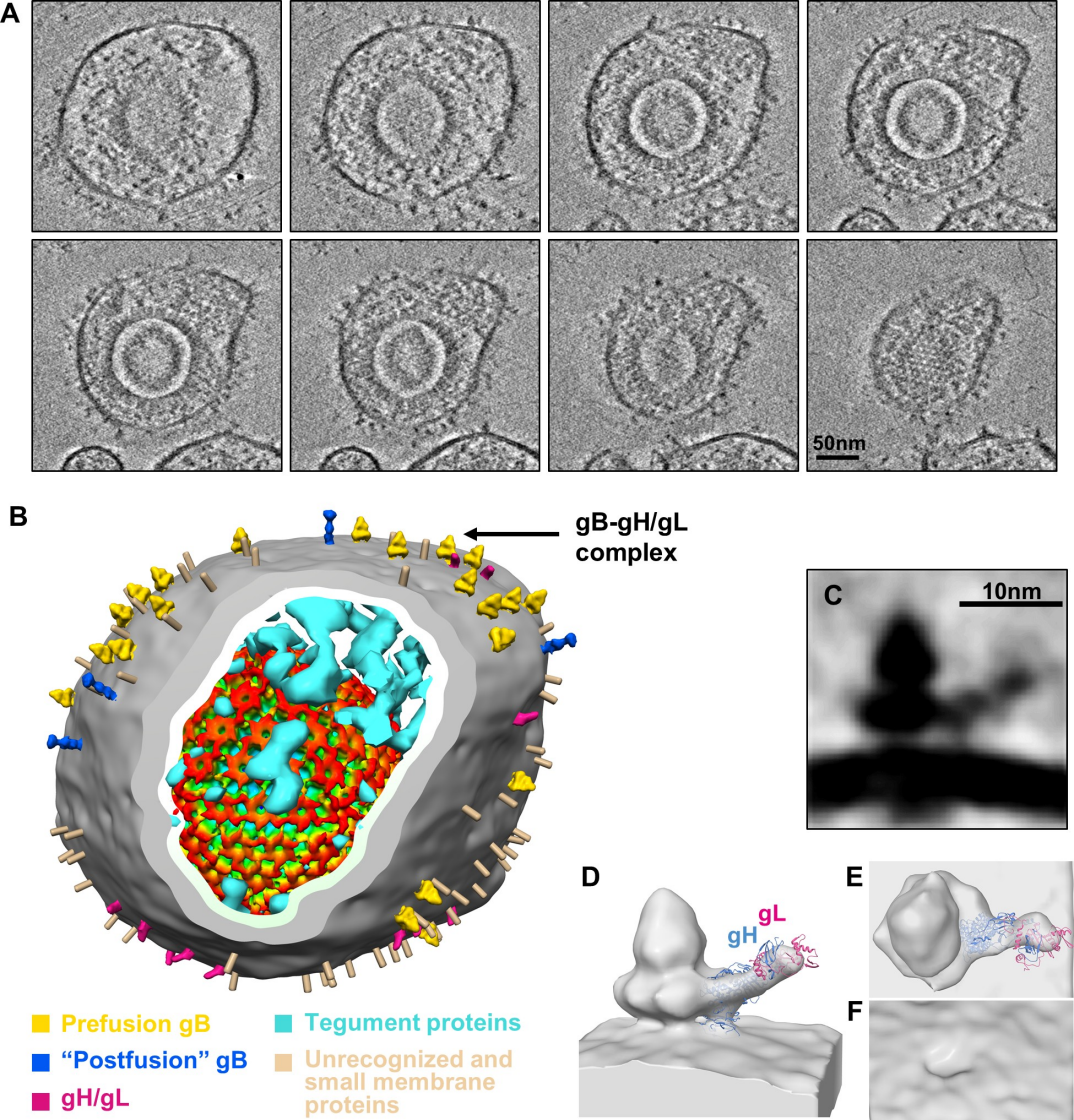
*In situ* structure of gH/gL complex adjacent to prefusion gB. (A) A series of slices in a tomogram showing a HCMV particle at different sections. (B) A 3D surface view from the HCMV particle in (A) with subtomographic average of prefusion and “postfusion” gB and gH/gL placed back on the viral envelope segmented from the tomogram. Black arrow indicates the gB/gH/gL complex. Unidentified glycoprotein densities are indicated as rods. Tegument proteins are shown as cyan densities. The recently published icosahedral reconstruction of capsid [11] was low-passed to 10Å, radially colored and placed back in its location. (C~F) The subtomographic average (C) showing a putative gH/gL complex adjacent to prefusion gB. The subtomographic average is also shown fitted with crystal structure of gH/gL (ribbon) (PDB: 5VOB) [17] in semitransparent surface viewed from side (D), top (E) and bottom (F).

Among herpesviruses, gB and gH/gL are highly conserved and known to form a fusion machinery for virus entry [40]. Previous biochemical studies have indicated that gH/gL regulated fusion activity of gB [41] and might form a complex with gB in virions on the basis of co-immunoprecipitation experiments [42]. Besides gB trimer densities mentioned above, “L”-shaped spikes were also observed protruding outwards from the viral envelope, which we interpret as gH/gL complexes on the basis of size and shape similarities to the gH/gL crystal structure [13, 17]. Moreover, among such “L”-shaped spikes, ~7% were observed to be in contact with the Christmas tree-shaped, prefusion gB trimer, forming a gB-gH/gL complex (Fig 6B and 6C), while others were unbound. No “postfusion” gB trimer have been observed involving in gB-gH/gL complex.

A subtomographic average was obtained by aligning and averaging 49 such gB-gH/gL complexes to investigate the contact sites between prefusion gB and gH/gL (Fig 6C–6F), with a resolution around 30Å reported by *calcFSC* in *PEET*. The HCMV gH/gL crystal structure [17] fits well in the “L”-shaped density in the subtomographic average (0.75 of the cross-correlation coefficient between the cryoET map and the model filtered to 30Å, Fig 6D and 6E). This fitting, together with the predicted domain arrangement in the prefusion gB structure (Fig 5H and 5I), reveals that DI of gB may contact the gH subunit of gH/gL (Fig 6D). The contact sites on gB and gH are consistent with the gH-binding site on HSV-1 gB suggested by blocking gH binding to gB with SS55 and SS56 antibodies (epitopes mapped to residues 153–363 of gB) (Fig 5H) [43] and the gB-binding sites on gH/gL suggested by anti-gH/gL antibody LP11 for HSV [13], respectively. Mutagenesis of gH cytotail has led to its proposed role of acting as a “wedge” to split the gB endodomain “clamp” to trigger gB ectodomain refolding [44]. Though the details of their interactions in the endodomain are yet to be resolved, this first observation of gH/gL complex making contact with prefusion gB *in situ* (Fig 6) supports the notion that receptor binding to gH/gL triggers transformation of gB from prefusion to postfusion conformation.

## Discussion

**Fig 7:**
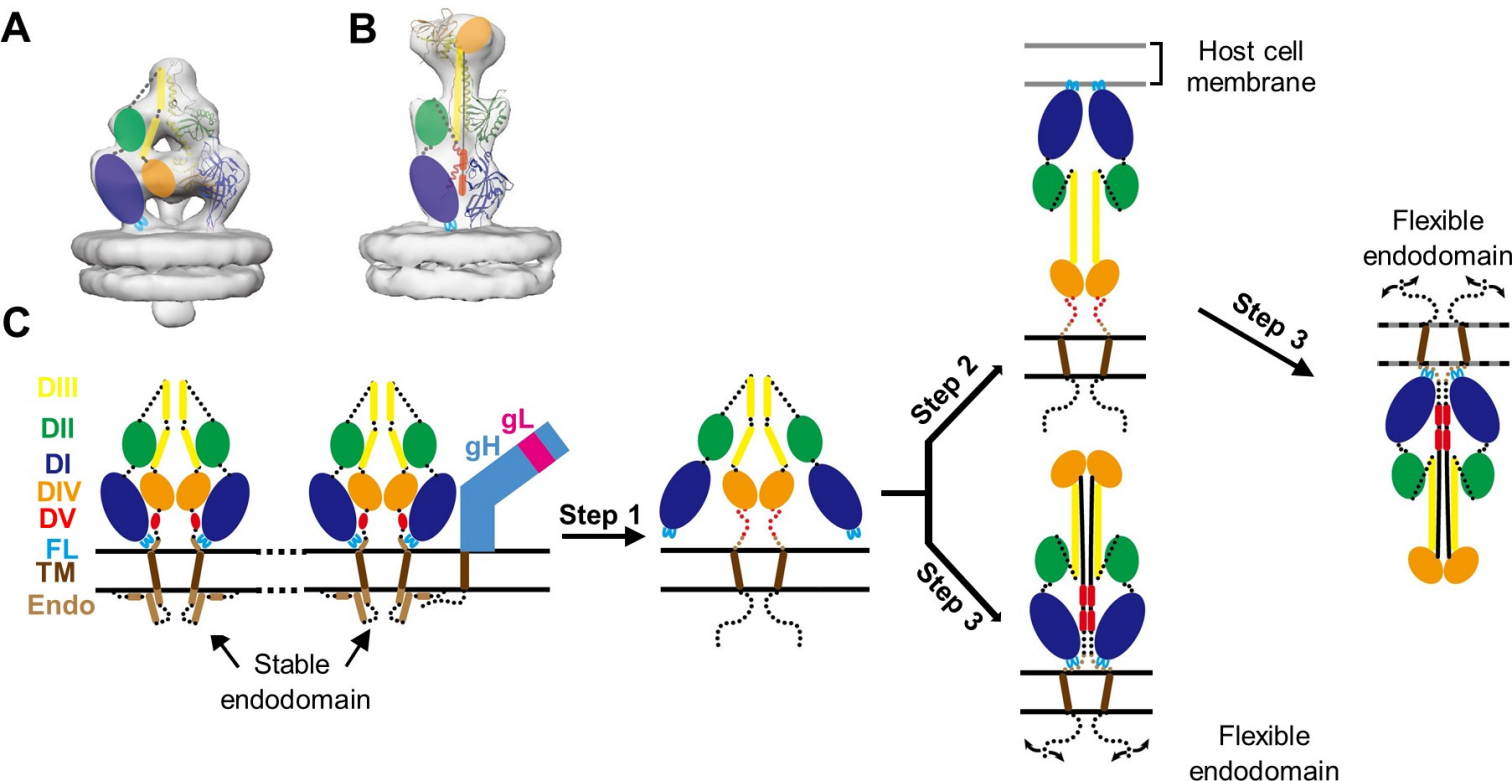
Schematic illustration of conformation changes of gB during membrane fusion. (A, B) Subtomographic averages of prefusion gB (A) with domains illustrated as in Fig 3 and “postfusion” gB (B) with domains colored as in [16]. (C) A working model of gB conformational change during membrane fusion. In step 1, destabilization of the endodomain of prefusion gB either by cytotail conformational changes following gH/gL receptor-binding or by other means (*e*.*g*., mechanical stress such as high-speed centrifugation during viral purification) triggers DI and DII to reorient, exposing the fusion loops on DI. Subsequently, the exposed fusion loops could make contact either with cell membrane in close proximity (in the case of receptor binding) (step 2) or with viral membrane. Finally (step 3), DV refolds into an extended form, transforming gB into its “postfusion” conformation: in the presence of cell membrane, the C-terminal part and the fusion loops come together and the membranes fuse; in the absence of cell membrane, the exposed fusion loops insert into the viral membrane.

Since the postfusion conformation of gB is energetically favorable and structurally more stable, it is not surprising that purified recombinant gB so far have all adopted the “postfusion” conformation [12, 16,45]. Therefore, imaging gB in its native, virion environment by cryoET seems to be the necessary approach to obtain the *in situ* structure in its metastable, prefusion conformation. However, a major challenge in interpreting *in situ* cryoET structures is the intrinsic poor contrast of tomographic reconstructions due to the use of low electron dose in order to avoid radiation damage to specimen. Poor contrast makes it difficult to identify different molecules or structures for subtomographic averaging. Normally for cellular tomography without phase plate, one could image with a large defocus value to achieve better contrast, aiding in distinguishing densities with different characteristics for subtomographic averaging. However, such approach only offers limited improvements in contrast (S1 Fig), and difficulties still exist in identifying the slender gB in postfusion conformation in our tomograms. This experience is consistent with two previous cryoET studies on HSV-1 gB structures, in which large defocus values were used to increase contrast to facilitate subsequent subtomographic averaging, yet the resulting structure either is at much lower resolution [26] than reported here or has led to controversial interpretations [27]. The greatly improved contrast afforded by VPP technology allowed the differentiation of various glycoprotein structures based on their characteristic appearances on the virion membrane (Fig 7A and 7B; S1 Fig). Therefore, cryoET with VPP offers a clear advantage in resolving structures of proteins in the native environments, enabling their identifications and subtomographic averaging to obtain structures of multi-functional states, as also demonstrated by the existence of two states of 26S proteasome inside neurons [31].

A vital step of herpesvirus infections is the fusion of viral and cell membranes, a complicated process involving at least three conserved proteins—gB, gH and gL. The *in situ* structures of gB at both prefusion and “postfusion” conformations reported here can shed lights on conformational changes of gB during membrane fusion and inform how herpesvirus entry into cell (Fig 7). Prior to fusion, gB needs to be maintained at its inactive, metastable prefusion conformation (Fig 7A). The maintenance of this metastable conformation possibly involves a properly-folded endodomain of gB, since removal of the endodomain caused gB ectodomain to adopt the postfusion conformation [46]. In addition, the direct observation in our cryoET structure of gB-gH/gL complex (Fig 6) and its isolation by co-immunoprecipitation [42] both suggest that the metastable ectodomain of gB might also be stabilized through the interaction with the ectodomain of gH subunit (Fig 7C). Host receptor-binding to gH/gL complex would trigger a conformational change in gH/gL cytotail and its dissociation from, and the destabilization of, the endodomain of gB, which in turn triggers the massive conformational changes of gB ectodomain to expose its fusion loops (step 1). Subsequently, DIII central helix extends, unfurling other domains and swinging the fusion loops to engage with the host membrane (step 2). Facilitated by the intrinsic fluidity in the plane of the membrane, the refolding of gB domains to the lower-energy, postfusion conformation, in which its ectodomain C-terminal end and the fusion loops must come together, leads to fusion of the two membranes and the release of viral DNA-containing capsid into cytoplasm (step 3). In the absence of receptor binding as in the situation of this study, mechanical stress to the membrane caused by such means as high-speed centrifugation could also destabilize the membrane-associated endodomain, triggering metastable prefusion gB to undergo the cascade of transformation events, possibly accompanied by the exposure of the fusion loops (step 1). Lacking host cell membrane, these events, with exposed fusion loops eventually encountering and inserting its hydrophobic moieties into the viral membrane, will be followed by refolding of other domains into the stable, “postfusion” conformation (step 3). Notably, the topology of the conformational change during step 2 to step 3 would preclude transiting from prefusion to postfusion conformation without breaking the three-fold symmetry. Indeed, monomeric intermediates of VSV G have been observed both in solution and on the surface of virions at intermediate pH conditions [22, 23].

In our model, the fusion loops of prefusion gB point to and are in close proximity to the viral membrane, possibly buried within a hydrophobic “mask” of MPR, which is attached to the C-terminal end of the gB ectodomain crystal structure. This membrane-proximal location of the gB fusion loops is the same as that based on the cryoET structure of the HSV-1 gB/anti-fusion loop 2-antibody at 5nm resolution [26] and is consistent with the fusion loop locations in all known atomic structures of classes I and III viral fusion proteins, including influenza HA [47], HIV env trimer [48], VSV G [21] and others [6]. Notably, our model is in stark contrast to the exposed fusion loops assigned to the membrane-distal tips of the “short-form” HSV-1 gB structures [27], which were obtained by cryoET of purified gB-containing vesicles. The ectodomain of the “short-form” vesicular HSV-1 gB structure is 15% shorter in height and 23% wider in diameter than that of our *in situ* HCMV gB structure, despite both sharing the Christmas tree shape (S5 Fig). Superposition of the domain assignment obtained by the hierarchical fitting approach [27] into the “short-form” HSV-1 gB structure shows that the densities projecting from the lower whorl of the Christmas tree-shaped trimer were unaccounted for (S5B Fig). Moreover, placing the same domain assignment into our *in situ* HCMV gB prefusion structure reveals that the fusion loops in this assignment are projecting out of the cryoET map, yet the leader density of the map is not accounted for (S5C Fig). When filtering the pseudoatomic model to 25Å, the cross-correlation coefficient is 0.74, as compared to 0.93 of our prefusion structure. We believe that an exposed fusion loop orientation of prefusion gB is unlikely for both chemical and biological reasons—exposed hydrophobic moieties are chemically unfavorable in solution and can lead to unproductive membrane insertion during infection. Indeed, the “short-form” HSV-1 gB structure was cautiously interpreted as an ambiguous “prefusion and/or intermediate” conformation [27], probably to reconcile these contradictory considerations.

Secondary structure prediction indicates that the endodomain is helix-rich (~50%) (Fig 4A). Our results suggest that gB endodomain undergoes significant conformational changes, from prominently visible/stable in the prefusion structure (Fig 3I and 3K), to invisible/flexible in the “postfusion” structure (Fig 3C and 3E). Proteolysis and circular dichroism analyses of the endodomain of the highly homologous HSV-1 gB posit that gB endodomain clamps the viral membrane and stabilizes gB in its prefusion conformation [44,49]. This proposed model is supported by studies on truncation and substitution mutations in endodomain [44,46]. The structured endodomain resolved in the recent crystal structure of full-length gB was thought to be similar to that in prefusion gB [24]. Detergent solubilization of the membrane may be responsible for the postfusion conformation of its ectodomain. Our observation of the endodomain structure of HCMV gB changing from a stable, prefusion conformation (Fig 3I) to a flexible, postfusion conformation (Fig 3E) is consistent with its proposed role in stabilization of gB prefusion conformation on native viral membrane [24].

## Materials and methods

### HCMV virion preparation

Human fibroblast MRC-5 cells (ATCC) were cultured in Eagle's Minimum Essential Medium (EMEM, ATCC) with 10% fetal bovine serum (FBS, Omega scientific: FB-11). Cells were grown in T-175 cm2 flasks to 90% confluence and infected with HCMV strain AD169 (ATCC, Rockville, MD) at a multiplicity of infection (MOI) of 0.1–0.5, and incubated for about 7 days. Once the cells showed 100% cytopathic effect, the media were collected and centrifuged at 10,000 g for 15 min to remove cells and large cell debris. The clarified supernatant was collected and centrifuged at 60, 000 g for 1 hour to pellet HCMV virions. Pellets were resuspended in 20mM phosphate buffered saline (PBS, pH 7.4), loaded on a 15%–50% (w/w) sucrose density gradient, and centrifuged at 60,000 g for 1 hr. After the density gradient centrifugation, three light-scattering bands were observed in the density gradient: top, middle and bottom. The middle band contained both HCMV virions and NIEPs (particles with intact viral envelopes as judged by negative-staining EM) and was collected, diluted in PBS and then centrifuged at 60,000 g for 1 hour. The final pellet was resuspended in PBS for further cryoET sample preparation.

### VSV virion preparation

VSV virion (Indiana serotype, San Juan strain) samples were produced as previously described [50]. Particularly, the inoculum was passaged multiple times in Hela cells with a very low multiplicity of infection (MOI), 0.001, to suppress the truncated defective-interference particles. The full VSV particles were isolated in a sucrose gradient and the final inoculum was also plaque-purified in Hela cells. We then pelleted the VSV virions at 30,000g for 2 hours and resuspended them in PBS. The stock was subjected to another low speed centrifugation at 12,000g for 5min in a desktop centrifuge to remove large aggregates. After resuspension, the pellets were banded on a 10ml density gradient containing 0–50% potassium tartrate and 30–0% glycerol. The virions-containing band was collected, diluted in PBS, pelleted at 30,000g for 2 hours, resuspended in PBS and kept in 4°C refrigerator for further cryoET sample preparation.

### CryoET sample preparation and data collection

An aliquot of 2.5 μl of the sample mixed with 5-nm diameter gold beads were applied onto freshly glow-discharged Quantifoil Holey Carbon Grids. Grids were blotted and plunge-frozen in liquid ethane cooled by liquid nitrogen using an FEI Mark IV Vitrobot cryo-sample plunger and were stored in liquid nitrogen before subsequent usage. CryoEM imaging and cryoET tilt series acquisition were performed with *SerialEM* [51] on an FEI Titan Krios 300kV transmission electron microscope equipped with a Gatan imaging filter (GIF), a Gatan K2 Summit direct electron detector, and with or without a Volta phase plate (VPP). Tilt series were recorded by tilting the specimen covering the angular range of -66° to +60° (starting tilt from -48° to +60°, then from -50° to -66°) with 2° or 3° interval, with a nominal magnification of x53,000 (corresponding to a calibrated pixel size of 2.6 Å) and a cumulative electron dose of 100~110 e^-^/Å^2^. Exposure time was multiplied by a factor of the square root of 1/cosα (in which α = tilt angle), and the exposure time at 0° was set at 1.2s for the tilt step-size of 2° or 1.6s for the tilt step-size of 3°. Movies were recorded with the frame rate of 0.2 frame/s on a Gatan K2 Summit direct electron detector operated in counting mode with the dose rate of 8–10 e^-^/pixel/s. An energy filter slit of 20 eV was chosen for the GIF. For imaging with VPP, defocus value was targeted at -0.6μm. Note, one of the benefits of using a phase plate is that the CTF is insensitive to the sign of the defocus value being negative (underfocus) or positive (overfocus) [52]. VPP was advanced to a new position every tilt series, followed by a 2 min waiting for stabilization, and pre-conditioned by electron illumination with a total dose of 12 nC for 60s to achieve a phase shift of ~54° as previously described [53]. For tilt series obtained without VPP, the defocus value was maintained at around -4μm while other imaging parameters were kept the same as those for the tilt series with VPP.

### 3D reconstruction

Frames in each movie of the raw tilt series were aligned, drift-corrected and averaged with *Motioncorr* [54] to produce a single image for each tilt angle. Both sets of tilt series, collected with and without VPP, were reconstructed with *IMOD* 4.8 software package [55] in the following six steps. All images in a tilt series were coarsely aligned by cross-correlation (step 1) and then finely aligned by tracking selected gold fiducial beads (step 2). The positions of each bead in all images of the tilt series were fitted into a specimen-movements mathematical model, resulting in a series of predicted positions. The mean residual error (mean distance between the actual and predicted positions) was recorded to facilitate bead tracking and poorly-modeled-bead fixing (step 3). With the boundary box reset and the tilt axis readjusted (step 4), images were realigned (step 5). Finally, two tomograms were generated by weighted back projection and simultaneous iterative reconstruction technique (SIRT) method, respectively (step 6). For data collected without VPP, contrast transfer function (CTF) was corrected with the *ctfphaseflip* program [56] of IMOD in step5. The defocus value for each image in one tilt series was determined by *CTFTILT* [57], and the estimated defocus value of each image was used as input for *ctfphaseflip*.

### Subtomographic averaging

Subtomographic averaging was performed using *PEET* 1.11 [58, 59]. High contrast SIRT tomograms were 4× binned by the *binvol* program of *IMOD* to facilitate particle picking. Particles were picked manually in *IMOD* as follows. For distinct conformations of VSV G and HCMV gB on viral envelope, two points (*head* and *tail*) in one contour were used to define one particle (glycoprotein)—*head* is the membrane-proximal end of the protrusion density while *tail* is the membrane-distal end. An initial *motive* list file, a RotAxes file and three model files containing the coordinates of *head*, *centroid* and *tail* for each particle were generated by *stalkInit* in *PEET*. In total, we manually picked 337 long-form particles from 5 VPP tomograms of VSV, and 350 columnar tree-shaped particles and 886 Christmas tree-shaped particles from 11 VPP tomograms of HCMV. Besides, 637 Christmas tree-shaped particles were picked from 28 non-VPP tomograms, averaged either alone or together with those from the VPP tomograms for prefusion gB.

For the reconstruction of the long-form VSV G, subtomographic averaging was performed first with 4× binned SIRT tomograms using the sum of all particles as the initial reference. Through *stalkInit*, each particle’s tilt orientation (*i*.*e*., the axes normal to the membrane) was already coarsely aligned to Y axis, but its twist orientation (*i*.*e*., the angle around the axis) was randomized. Therefore, in the first refinement cycle, we set the angular search range 180° max (-180° to 180°) with 9° step in Phi (Y axis), and 5° (-5° to 5°) max with 1° step in both Theta (Z axis) and Psi (X axis), and search distance 3 pixels along all three axes. Due to the known symmetry of postfusion VSV G, the resulting averaged structure was then trimerized and used as the reference of the next refinement cycle. The trimerized structure was the sum of each refined particle and its two symmetrical copies—the two symmetrical copies have the same position and tilt orientation as the refined particle, but twist orientation differed by either 120° or 240°. For subsequent refinement cycles, the newly trimerized structure from the last refinement cycle was used as reference, with both angular and distance search ranges narrowing down gradually. After four refinement cycles, the averaged structure converged based on no further improvement in resolution. The following refinement cycles were performed with 2× binned tomograms reconstructed by weighted back projection, after up-sampling (generations of 2× binned model files and updates of corresponding motive list files from the latest refinement cycle), with small search distance range (4 pixels) and narrow angular search range (-20° to 20°). The reference was updated from the averaged structure of the last refinement cycle (trimerized). For particles with distance of <1 pixel and twist angle difference of <1°, the one representative with lower cross-correlation coefficient was treated as duplicate particle and removed during the refinement. The averaged structure, contributed by 330 particles, converged after eight refinement cycles and was filtered to the final resolution, calculated by *calcFSC* in *PEET* based on the 0.143 FSC criterion.

Reconstructions of columnar tree-shaped and Christmas tree-shaped particles on HCMV envelope followed the same refinement procedure as the reconstruction of long-form VSV G, except that trimerization was only applied after three-fold symmetry became apparent in the averaged structures. With the removal of duplicate particles, the final averaged structures of the postfusion (columnar tree-shaped) and prefusion (Christmas tree-shaped) conformations were obtained from 350 particles and 1509 particles, respectively. Furthermore, gold-standard FSC calculations for the structures were performed afterwards by splitting the original dataset of each conformation into two independent groups. The same refinement procedure used above was applied to the two newly-generated groups independently. Upon the convergence of the averaged structures, FSC were calculated by *calcUnbiasedFSC* in *PEET* (S3A Fig.).

For the reconstruction of the short-form VSV G, 65 particles were manually picked from five tomograms with single point to define the centroid position. Each particle was manually rotated around X, Y, Z axes to a similar orientation (both the tilt orientation and twist angle) in *IMOD* slicer window. By *slicer2MOTL* in *PEET*, the initial motive list files for subtomographic averaging were generated from the corresponding X, Y, Z rotation degrees. For the Angular Search Range, small search range was set during all seven refinement cycles. The final subtomographic average was Gaussian filtered with width 7 using the “volume filter” tool in UCSF *Chimera* [60]. Due to the limited number of particles (49 particles), HCMV gB-gH/gL complex was reconstructed with the same strategy above.

### 3D visualization

We used *IMOD* [61] to visualize reconstructed tomograms and UCSF *Chimera* to visualize the subtomographic averages in three dimensions. The crystal structures of prefusion VSV G (PDB: 5I2S) [21], postfusion VSV G (PDB: 5I2M) [20], HCMV postfusion gB (PDB: 5CXF) [16] and gH/gL part from HCMV pentamer (PDB: 5VOB) [17] were fitted into subtomographic averages of prefusion G, postfusion G, postfusion gB and gB-gH/gL complex, respectively, with the tool *fit in map* in *Chimera*. Segmentation and surface rendering for the membrane and tegument proteins were done by the tools *volume tracer* and *color zone* in *Chimera*. All membrane glycoproteins were placed back on the viral membrane according to their locations in the original tomogram. A published structure of HCMV capsid with inner tegument protein [11] was filtered to 10 Å and placed back at the same position of the capsid in tomogram.

### Domain modeling and structure prediction

As outlined below, we employed a combination of initial manual fitting of known domain structures, followed by simulation with MDFF program [38] to generate a gB prefusion model based on our cryoET prefusion gB trimer density map and the existing gB ectodomain postfusion crystal structure (PDB: 5CXF) [16]. First, the ectodomain in the subtomographic averaged density map of prefusion gB trimer was segmented out and its symmetric axis obtained with *Chimera*’s “volume eraser” tool and “measure symmetry” command, respectively. Second, *Chimera*’s “fitmap” command with “global search” and 15Å-resolution options was used to refine 1000 initial random DI placements, resulting in 28 refined fitted positions, each with a correlation coefficient (between the fitted model and the density map) and a “clash volume fraction” value (between symmetry-related copies). We chose the fitted position with the largest fitting score, defined as the correlation coefficient subtracted by the “clash volume fraction” penalty value (Fig 5A). Third, we obtained our initial DIII by computationally mutating the DIII model from the existing hypothetic model of EBV prefusion gB [14], as it is known to differ substantially from its postfusion conformation for both herpesvirus gB [14, 62] and homologous VSV G [21]. Compared to that in the postfusion gB, the central helix α4 in DIII in the prefusion gB is bent in order to fit into the top of the Christmas tree-shaped density. This bent varies from only ~30° in our proposed HCMV gB prefusion structure to ~90° in VSV G (Fig 5D) [21] and ~180° in influenza HA [47] and HIV env [48]. This DIII model, and the models of DII and DIV from the gB postfusion crystal structure were manually fitted as rigid bodies into our prefusion gB trimer cryoET density to produce a composite model with the above obtained DI trimer model by referencing the prefusion VSV G crystal structure. Connecting loops were then added to this composite model through the *Modloop* server [63]. Fourth, the resulting trimer model was used as the initial model for MDFF simulations [38] with grid force scale of 0.3. Secondary structure, *cis* peptide and chirality restraints were imposed during MDFF simulations. Simulations were performed with NAMD 2.12 [64], using the CHARMM36 force field with CMAP corrections [65].

Secondary structures for residues 707–906 of gB were predicted with *Phyre2* [66].

## Supporting information

S1 Fig Comparison of tomograms obtained with and without VPP. (A~C) A slice (A) and zoom-in envelope regions (B, C) of a tomogram reconstructed from tilt series obtained with VPP, showing greatly improved contrast that is sufficient to distinguish columnar tree-shaped (“postfusion”) gB (yellow arrows in B) from the Christmas tree-shaped (prefusion) gB (red arrows in C).
(D~F) A slice (D) and zoom-in envelope regions (E, F) of a tomogram reconstructed from tilt series obtained without VPP, showing the relatively poor contrast and great ambiguity to distinguish columnar tree-shaped (“postfusion”) gB (yellow arrow in E) from the Christmas tree-shaped (prefusion) gB (red arrow in F). Consequently, significantly more tilt series without VPP than with VPP had to been recorded in order to obtain similar number of particles for subtomographic averaging.
(TIF)

S2 Fig Subtomographic averages of gB without imposing symmetry. (A, B) Subtomographic averages of gB in its postfusion conformation without imposing symmetry viewed from side (A) and top (B).
(C, D) Subtomographic averages of gB in its prefusion conformation without imposing symmetry viewed from side (C) and top (D).
(TIF)

S3 Fig Fourier shell correlation (FSC) analyses and resolution comparisons. (A) FSC coefficients as a function of spatial frequency for the gold-standard resolution determined for final subtomographic averages of prefusion (black) and “postfusion” (red) gB trimers.
(B) FSC coefficients as a function of spatial frequency between subtomographic averages of prefusion gB trimers obtained with VPP and without VPP. For the average obtained without VPP, CTF correction is necessary as indicated by the negative correlation coefficients in the range from 1/26 Å^-1^ to 1/20 Å^-1^ spatial frequencies.
(TIF)

S4 Fig Tomograms and subtomographic averages from tilt series obtained without VPP. (A, B) Comparison of corresponding slices from CTF-uncorrected (A) and CTF-corrected (B) tomograms. The viral envelope region of the particle indicated by the dashed boxes in (A, red) and (B, yellow) are enlarged, showing that the membrane bilayer is better resolved after CTF correction in the yellow zoom-in inset.
(C, D) Subtomographic average of the Christmas tree-shaped densities (yellow) and associated membrane bilayer (blue) viewed from side, top and slanted bottom. (C) is obtained without CTF-correction and (D) is with CTF-correction.
(E) FSC coefficients as a function of spatial frequency between subtomographic averages of prefusion gB trimers obtained with CTF correction and without CTF correction. For the subtomographic average obtained without VPP, CTF correction is necessary as indicated by the negative correlation coefficients (grey zone) for some spatial frequencies.
(TIF)

S5 Fig Direct comparisons of the averaged map and fitted pseudoatomic model between prefusion HCMV gB and previous “short-form” HSV-1 gB. (A) Christmas tree-shaped prefusion gB on HCMV virion (this study), reviewed from its side and top, as in Figs 3 and 5.
(B) “Short-form” gB of HSV-1 [27] showing as in (A), colored surface (upper panel) and superposition (lower panel) of semitransparent density (gray) and fitted domains (ribbons). Red dotted ellipses indicate the densities that were unaccounted for by the fitted psedoatomic model.
(C) Superposition of the proposed HSV-1 gB domain (DI and DII) arrangement from [27] into the density of the prefusion gB trimer on HCMV virion (this study) showing mismatch, which is indicated by red dotted ellipses.
(TIF)

S1 Movie An example of aligned tilt series obtained with VPP. (Scale bar: 100nm.)
(AVI)

S2 Movie Slices through a tomogram reconstructed by simultaneous iterative reconstruction technique (SIRT) from tilt series in S1 Movie. (Scale bar: 100nm.)
(AVI)

S3 Movie An example of aligned tilt series obtained without VPP. (Scale bar: 100nm.)
(AVI)

S4 Movie Slices through a tomogram reconstructed by SIRT from tilt series in S3 Movie. (Scale bar: 100nm.)
(AVI)

S5 Movie Surface rendering of the subtomographic average of “postfusion” gB (yellow) and associated membrane bilayer (blue). (AVI)

S6 Movie Surface rendering of the subtomographic average of prefusion gB (yellow) and associated membrane bilayer (blue). (AVI)

S7 Movie MDFF-simulated prefusion gB structure, colored as [16], is superimposed with two other symmetric copies (gray ribbon) in the subtomographic average of the Christmas tree-shaped density (semi-transparent gray). (AVI)
