# Supplementary figures and images for: Different functional states of fusion protein gB revealed on human cytomegalovirus by cryo electron tomography with Volta phase plate
Source: PLoS Pathog. 2018 Dec 3;14(12):e1007452. doi: 10.1371/journal.ppat.1007452 (PMC6307773; doi:10.1371/journal.ppat.1007452)

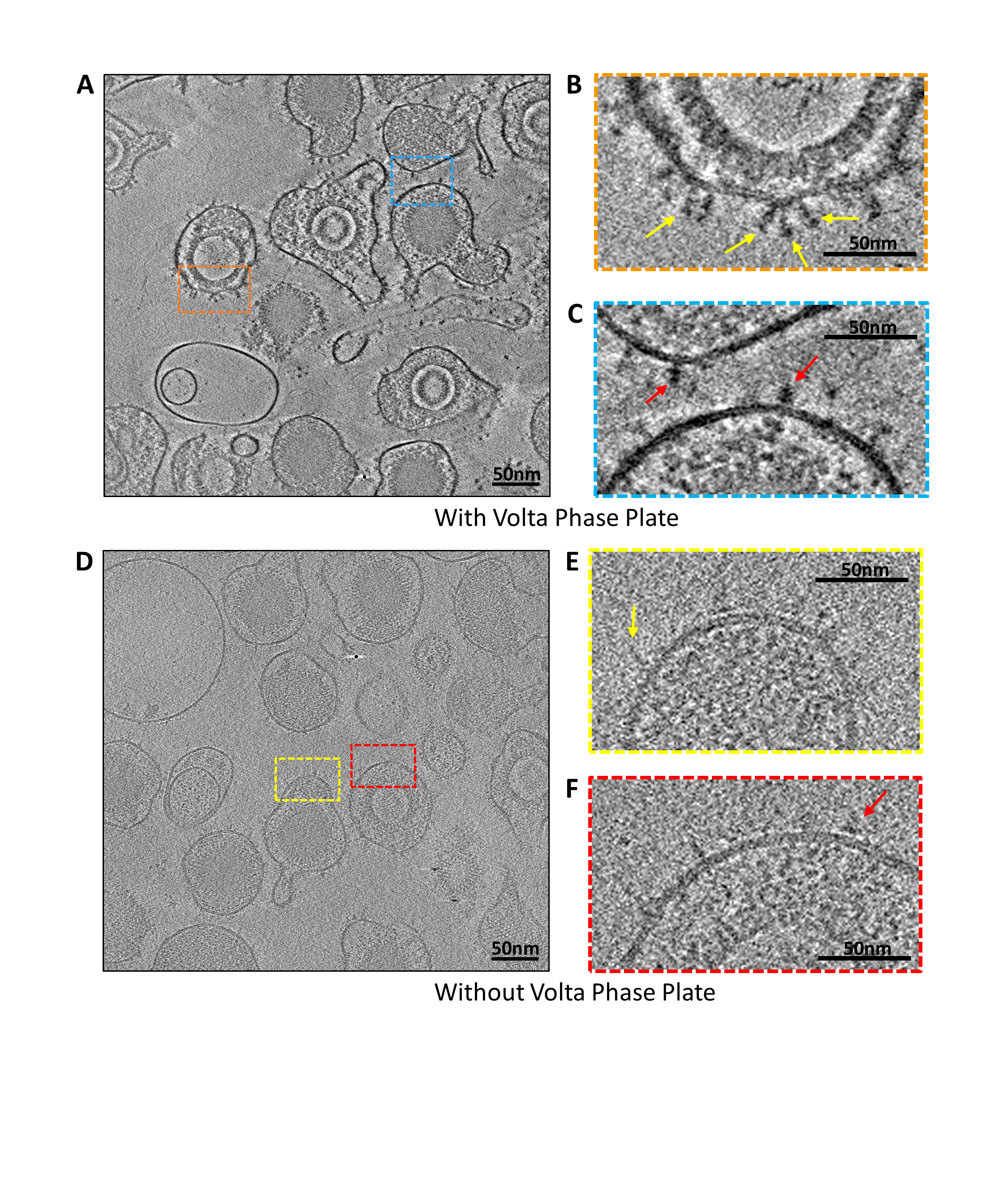

Supplement: S1 Fig — (A~C) A slice (A) and zoom-in envelope regions (B, C) of a tomogram reconstructed from tilt series obtained with VPP, showing greatly improved contrast that is sufficient to distinguish columnar tree-shaped (“postfusion”) gB (yellow arrows in B) from the Christmas tree-shaped (prefusion) gB (red arrows in C). (D~F) A slice (D) and zoom-in envelope regions (E, F) of a tomogram reconstructed from tilt series obtained without VPP, showing the relatively poor contrast and great ambiguity to distinguish columnar tree-shaped (“postfusion”) gB (yellow arrow in E) from the Christmas tree-shaped (prefusion) gB (red arrow in F). Consequently, significantly more tilt series without VPP than with VPP had to been recorded in order to obtain similar number of particles for subtomographic averaging. (TIF) [file ppat.1007452.s001.tif]

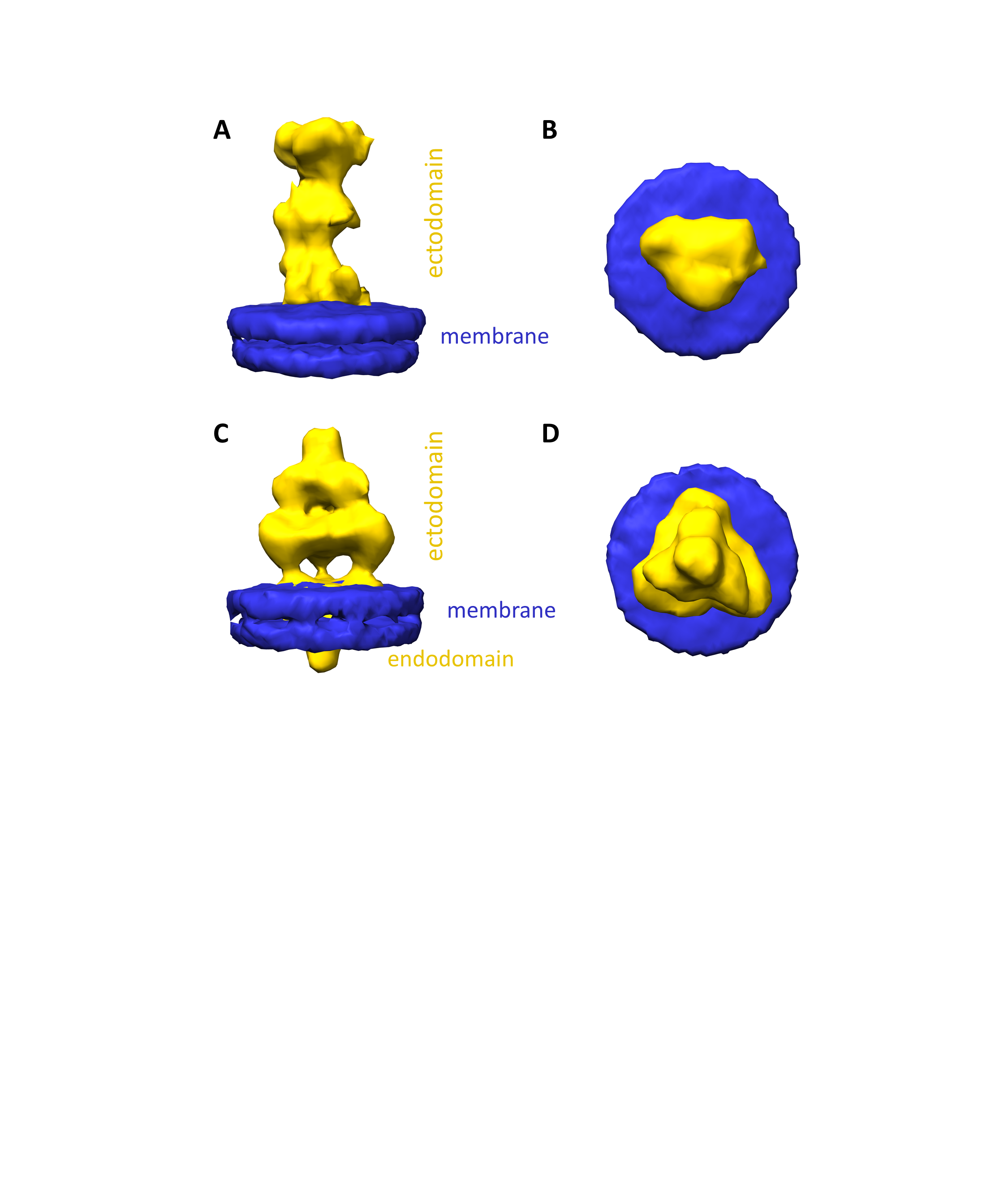

Supplement: S2 Fig — (A, B) Subtomographic averages of gB in its postfusion conformation without imposing symmetry viewed from side (A) and top (B). (C, D) Subtomographic averages of gB in its prefusion conformation without imposing symmetry viewed from side (C) and top (D). (TIF) [file ppat.1007452.s002.tif]

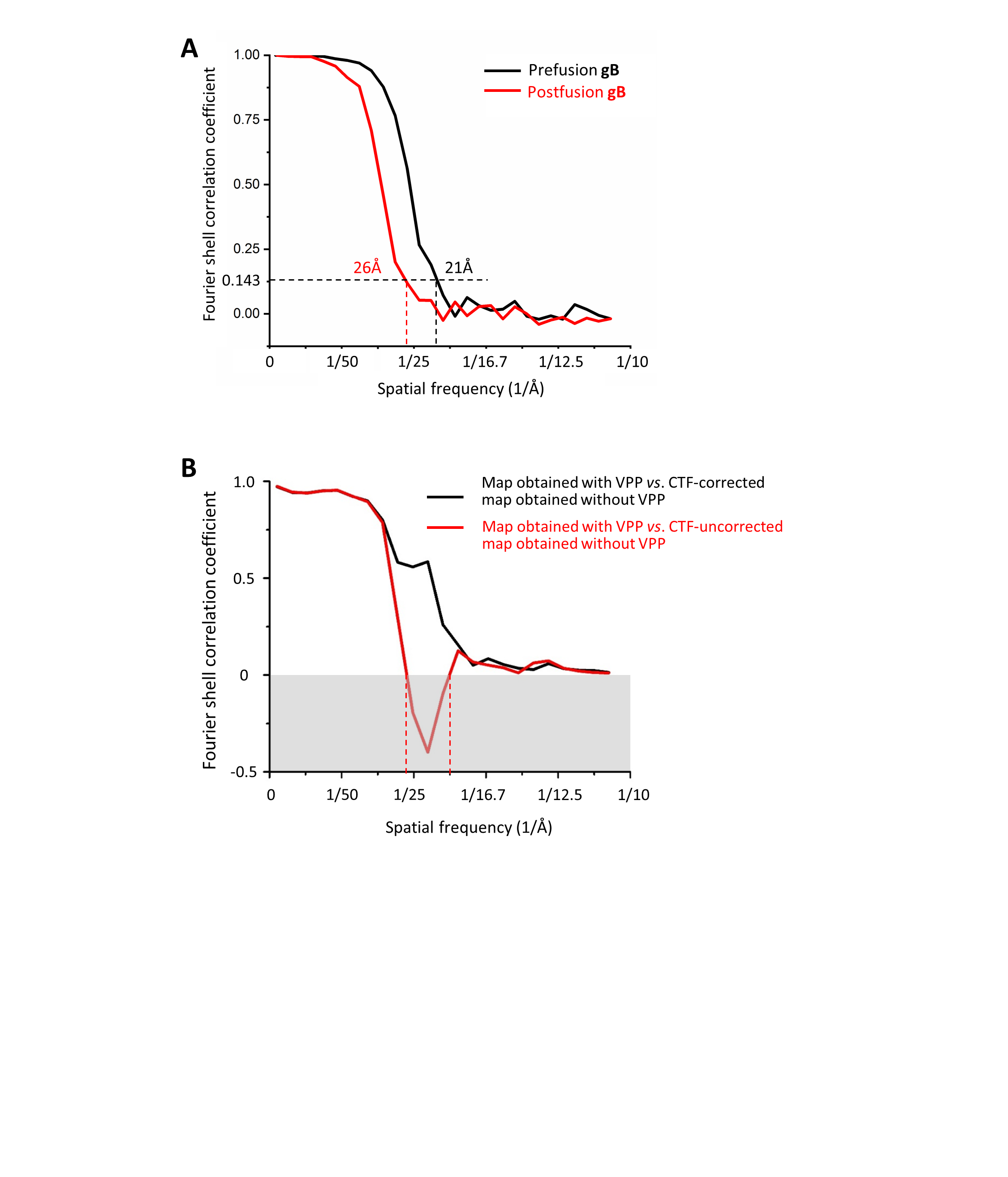

Supplement: S3 Fig — (A) FSC coefficients as a function of spatial frequency for the gold-standard resolution determined for final subtomographic averages of prefusion (black) and “postfusion” (red) gB trimers. (B) FSC coefficients as a function of spatial frequency between subtomographic averages of prefusion gB trimers obtained with VPP and without VPP. For the average obtained without VPP, CTF correction is necessary as indicated by the negative correlation coefficients in the range from 1/26 Å-1 to 1/20 Å-1 spatial frequencies. (TIF) [file ppat.1007452.s003.tif]

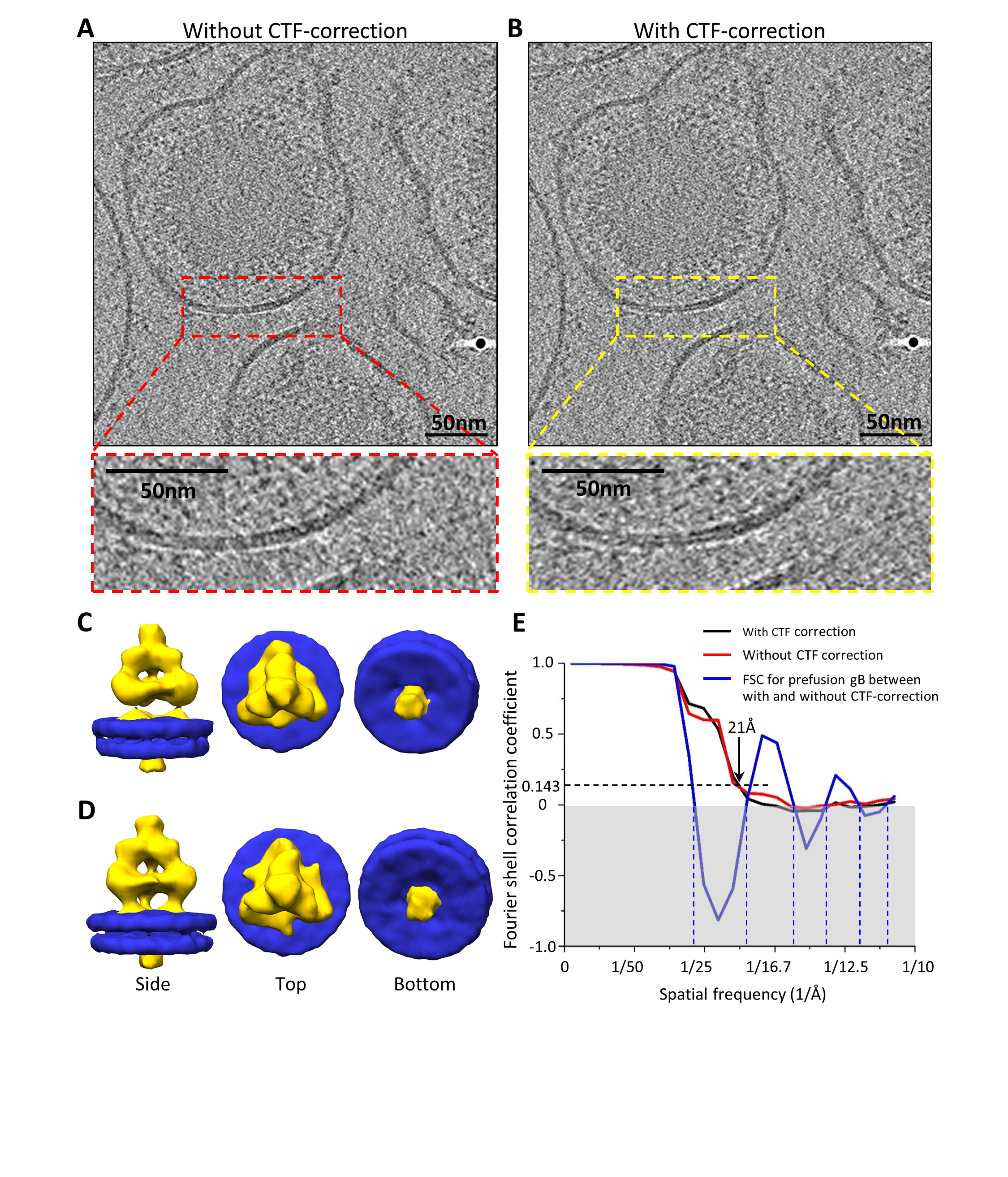

Supplement: S4 Fig — (A, B) Comparison of corresponding slices from CTF-uncorrected (A) and CTF-corrected (B) tomograms. The viral envelope region of the particle indicated by the dashed boxes in (A, red) and (B, yellow) are enlarged, showing that the membrane bilayer is better resolved after CTF correction in the yellow zoom-in inset. (C, D) Subtomographic average of the Christmas tree-shaped densities (yellow) and associated membrane bilayer (blue) viewed from side, top and slanted bottom. (C) is obtained without CTF-correction and (D) is with CTF-correction. (E) FSC coefficients as a function of spatial frequency between subtomographic averages of prefusion gB trimers obtained with CTF correction and without CTF correction. For the subtomographic average obtained without VPP, CTF correction is necessary as indicated by the negative correlation coefficients (grey zone) for some spatial frequencies. (TIF) [file ppat.1007452.s004.tif]

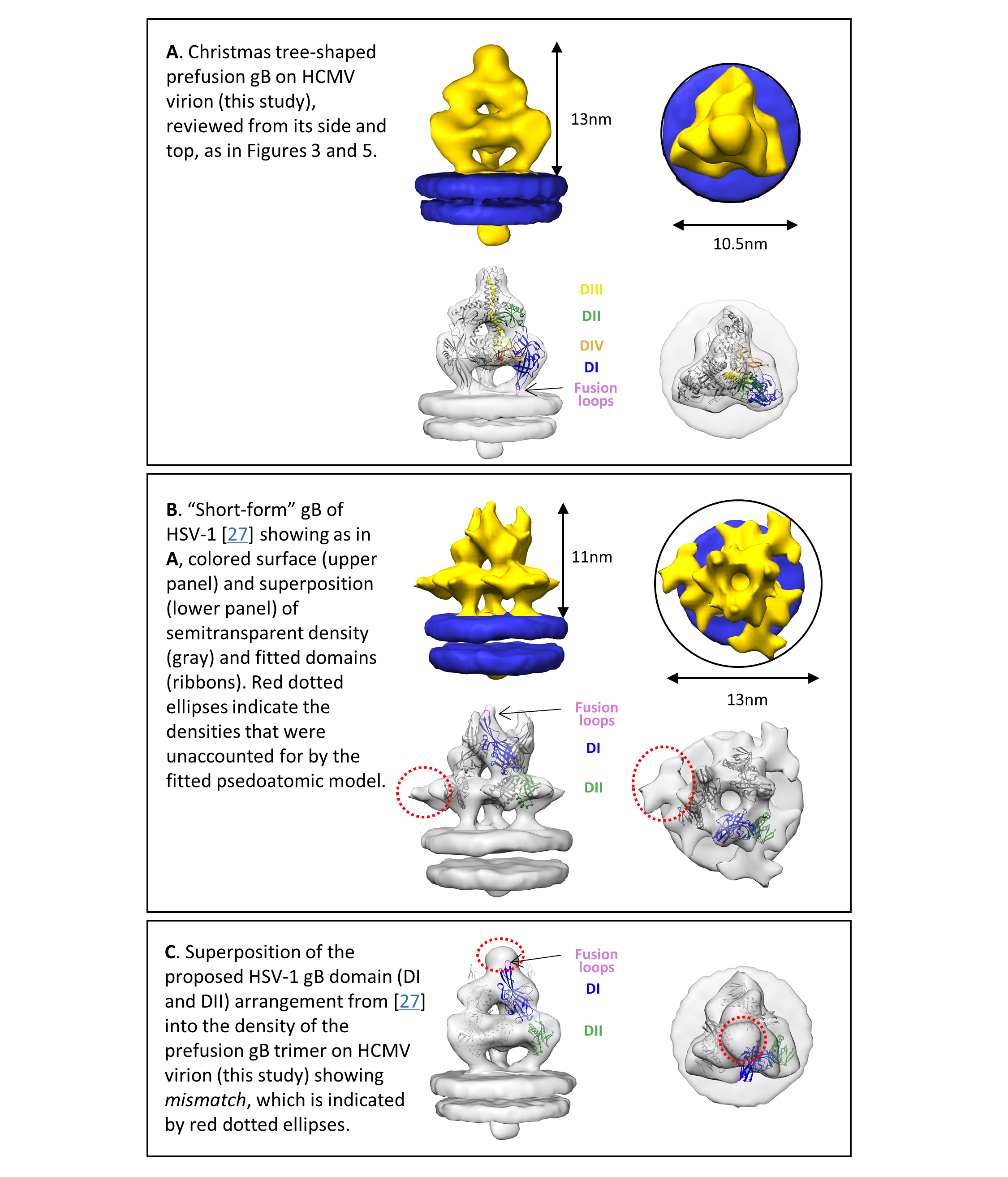

Supplement: S5 Fig — (A) Christmas tree-shaped prefusion gB on HCMV virion (this study), reviewed from its side and top, as in Figs 3 and 5. (B) “Short-form” gB of HSV-1 [27] showing as in (A), colored surface (upper panel) and superposition (lower panel) of semitransparent density (gray) and fitted domains (ribbons). Red dotted ellipses indicate the densities that were unaccounted for by the fitted psedoatomic model. (C) Superposition of the proposed HSV-1 gB domain (DI and DII) arrangement from [27] into the density of the prefusion gB trimer on HCMV virion (this study) showing mismatch, which is indicated by red dotted ellipses. (TIF) [file ppat.1007452.s005.tif]
